# Experiences of female clients in the assisted reproductive technology process in Nigeria

**DOI:** 10.4314/ahs.v23i2.76

**Published:** 2023-06

**Authors:** Ijeoma V Ezeome, Simisola O Akintola, Ayodele S Jegede

**Affiliations:** 1 Department of Bioethics, Faculty of Multidisciplinary Studies and Faculty of Clinical Sciences, University of Ibadan, Ibadan Nigeria; 2 Department of Obstetrics & Gynaecology, College of Medicine, Faculty of Medical Sciences, University of Nigeria, Ituku-Ozalla Campus, Enugu, Nigeria; 3 Department of Private & Business Law, Faculty of Law, University of Ibadan, Nigeria; 4 Department of Sociology, Faculty of the Social Sciences, University of Ibadan, Nigeria

**Keywords:** Experience, clients, assisted reproductive technology

## Abstract

**Background:**

Though assisted reproductive technology (ART) has been a source of hope for many infertile couples, it is known to be associated with physical, social, psychological, and ethical challenges in different settings.

**Objective:**

The study objective was to explore the experiences of female clients who accessed services in ART clinics in Nigeria.

**Methods:**

An exploratory qualitative design, using snowballing technique was employed. It involved in-depth interviews of eight women who had gone through the assisted conception process, using an interview guide. Data collection was from November 2019 to January 2020. Results: The age range of the participants was 30 – 52 years with a mean of 41.88 years. Majority of the clients presented at advanced age to their ART providers because of lack of funds and ignorance. Other challenges were suboptimal risk disclosure, high cost of services, feelings of shame and stigmatization during and after the process.

**Conclusion:**

Cost of ART remain unaffordable to most Nigerians hence the need for government subsidization and in-corporation of ART services into the National Health Insurance Scheme. Additionally, lack of adequate information disclosure, shared decision-making and gendered suffering are areas of great concern among the study participants. A national regulatory guideline will ensure uniform international standard of care.

## Introduction

Infertility is recognized by the World health Organization as a global public health issue 1. Worldwide, it is estimated that the absolute number of couples affected by infertility increased from forty-two million in 1990 to forty-eight and a half million in 2010 2. Among the various regions in the world, South East Asia and Sub-Saharan African countries have the highest prevalence of infertility where infection-related tubal damage is the commonest cause 3. Compared to Western societies the consequences of involuntary childlessness are usually more pronounced particularly for women, leading to such consequences of marital demise, physical violence, emotional abuse, social exclusion, community exile, ineffective and iatrogenic therapies, poverty, old age insecurity, increased risk of HIV/AIDS, and death 4. Infertility in Nigeria has been reported to be as high as twenty-five percent among couples 5, much higher than the global prevalence of eight to twelve percent 6. Globally, one in every six couples will experience a fertility problem during their reproductive age 7

Over the past one and half decades, the use of ART has become commonplace 8, increasing at a rate of five to ten percent annually 9. Inspite of its having brought succour to many childless couples/individuals, the associated ethical, legal, and social challenges remain issues of great concern. This has led to the institution of regulatory guidelines in many developed countries. However, there are huge variations in the legislative framework governing ART across countries as it depends on sociocultural factors, political choices and religious beliefs 10. Though African countries have embraced advanced reproductive technologies mostly as a private-sector endeavour, only South Africa in Sub-Saharan Africa has a national regulatory guideline in place 11.

The first baby born through IVF in Nigeria was in Lagos University Teaching Hospital by Oladapo Ashiru and his team in 1989 though there were documented pregnancies in 1984 and 1986 12. Subsequently, fertility centres have arisen throughout the country with provision of almost all forms of assisted conception services. However, at present it remains a private sector endeavour, driven more by commerce rather than ethics 13. Despite the enormously high cost of setting up facilities, there has been a steady growth of ART service centers in Nigeria. Nigeria is yet to have a national regulation for ART practice though the Association for Fertility and Reproductive Health (AFRH) has recently instituted non-legally binding guidelines for its members 14. Previous studies in Nigeria have documented stakeholders' views on the ethical and social challenges of ART, implicating high cost of services 15, 16, inappropriate practices by ART practitioners 17, large number of unregistered practitioners of in-vitro fertilization^13^, encouraging a lot of quackery, with psychological problems and discrimination against children born through ART 18. However, there is a dearth of data on the experiences of the female clients who undergo these procedures. The aim of the present study was to explore the experiences of female clients accessing ART services in Nigeria.

## Methods

An exploratory research design was used for the study, with in-depth interviews of clients who have gone through the ART process, focusing on their experience. Ethical approval was obtained from the Research Ethics Committee of UI/UCH (NHREC/05/01/2008a) and from the Research Ethics Committee of University of Nigeria Teaching Hospital, Enugu (NHREC05/01/2008B-FWA00002458-IRB00002323) as part of an MSc Bioethics Dissertation on Practitioners' and Clients' Perspectives and Experiences on the Regulation of ART in Nigeria.

The study population were purposively selected to answer the research questions. Enugu State was selected because of easy accessibility to the researcher.

The ART clients were recruited by the snowballing technique, where an ART client who consented to the study gives the researcher the name of a potential participant in confidence, snowballing into as many clients as agree to take part in the study. This is because women are secretive of their use of ART due to the stigma attached to it in Nigeria. Inclusion criteria was being a female client who had an ART procedure within the last two years prior to the onset of the study and domiciled in Enugu, irrespective of ethnic, sociocultural or religious affiliations.

The draft research questions were administered to one married woman who has gone through the ART process within two years prior to the onset of the study. Her responses helped the researcher confirm that the research questions were easy to understand and led to refining of the questions where necessary.

The aim of the study was explained to the research participants selected, ensuring that they freely consented to take part in the study. They were informed that the questions asked was to elicit information that would help improve ART practice and the experiences of clients during the ART process. They were also made to know that they were free to answer the questions asked as truthfully as possible and that there were no right or wrong answers, all comments were welcome and would be kept strictly confidential. The lead investigator also made the research participants know that the interview was to be recorded on tape and notes taken by a research assistant. A written informed consent was obtained from all research participants. The fieldwork was conducted by the lead investigator and a trained assistant who recorded the responses and took notes. The interview was in English for educated research participants and Igbo for those that were not literate. However, most women living in urban Enugu where the study took place spoke and understood Pidgin English at the minimum. Data collection lasted three months from November 2019 to January 2020.

An interview guide (attached as an appendix) was used to collect information on their experiences of the ART process. It was intended to recruit a sample of ten ART clients. However, saturation point was reached at the eighth interview. The interview was carried out in the homes of clients or at designated places where the clients were most comfortable.

All tapes were reviewed at the end of each session to confirm adequacy. The notes taken were also reviewed after every interview to be sure that correct responses were recorded by going through some of the questions randomly with the respondents or participants. The recordings were transcribed verbatim. To confirm reliability, five percent of the tapes were re-transcribed by another person to ensure correctness of information obtained.

A clean set of all the interview transcripts was handed over to an independent coder, a senior researcher with good knowledge of qualitative research methodology for analysis with a copy of clear instructions about the framework method of data analysis to be implemented. Once the researcher completed data analysis of the same interviews to identify themes, a consensus discussion meeting was held with the independent coder to discuss the emerging issues from the data analysis process. The framework creates a new structure for the data (rather than the full original accounts given by participants) that is helpful to summarize/reduce the data in a way that can support answering the research questions 19

The researcher explored the experiences of the clients during their journey through the ART process on key ethical issues in ART such as decision making about the use of ART, access issues, informed consent, discrimination against children born through ART, gendered suffering (psychological problems and loss of self-esteem resulting from the use of ART), side effects of the technologies and the cost of accessing the procedures. The results are set out in themes, based on the study objective.

## Results

### Demographic characteristics

The participants were aged 30 - 52 years with a mean age of 41.88 years, while spouses were aged 40 - 60 years with a mean age of 52.8 years. The clients had infertility ranging from three to thirteen years prior to seeking ART treatment. Other demographic characteristics are as in the table below.

**Table 1:** Demographic characteristics

female client	age in years	occupation	education	religion	tribe	marriage type	spousal age in years	occupation of spouse	location of ART centre accessed	cycle at which success was achieved
1	52	Lab scientist	University	Anglican	Igbo	Monogamous	60	Lawyer	Lagos	5, no success yet
2	30	Immigration officer	University	Pentecostal	Igbo	Monogamous	40	Business	Enugu	1
3	48	Lab scientist	University	Roman Catholic	Igbo	Monogamous	58	Business	Abuja	3
4	37	Business	Secondary	Pentecostal	Igala	Monogamous	50	Army	Enugu	1
5	38	Business	Polytechnic	Roman Catholic	Igbo	Monogamous	48	Business	Abuja	1
6	44	Pharm rep	University	Roman Catholic	Igbo	Monogamous	55	Business	Port-Harcourt	1
7	43	Business	Secondary	Pentecostal	Hausa	Monogamous	56	Army	Enugu	2
8	43	Medical doctor	University	Roman Catholic	Igbo	Monogamous	53	Medical doc.	Enugu	7

The major themes that emerged from the study were advanced age at presentation for ART, lack of information on how to access ART clinics, religious beliefs, high cost of ART, poor information disclosure, absence of shared decision-making, challenges with roads, and gendered suffering.

### Advanced age at presentation

Many of the clients, at presentation to the ART clinic, were on the average, above forty years of age. This was due to various reasons ranging from lack of financial resources, lack of referral from primary physician, and ignorance on how to access the needed care.

Respondent No. 3 said:

We have been married for six years. After two years of marriage without conception, we went to a doctor who told us there was nothing wrong after doing a series of tests on both of us. The doctor then gave me some drugs over a three-month period, which he said will help me ovulate, but we had no success. We kept moving from one doctor to the other till we got to know about ART from a relative. [forty-eight years old laboratory scientist, Igbo]

This was corroborated by respondent No. 1:

We went to hospital after two years of marriage with no pregnancy, but the doctor found nothing wrong with either of us. He told us to keep trying naturally. He even discouraged us from going for ART when we suggested it. It was after ten years of infertility that we eventually accessed ART [fifty-two years old laboratory scientist, Igbo]

### Information on how to access ART clinic

Most of the clients lacked information on how to go about accessing ART care, leaving them at risk of being exploited by unscrupulous individuals. Some of the participants got information from relatives and acquaintances. According to respondent No. 7,

As head of the Army Officers' Wives Association, a lady who had started consulting for ART asked for permission to attend her egg retrieval session. I told her I also had infertility problem and she encouraged me to come to the centre she was attending because of its high success rate [forty-three years old businesswoman, Hausa]

Similarly, respondent No 2 said:

A friend of mine in Church told me about the centre I consulted. She said she had a relative who was successful [thirty years old immigration officer, Igbo]

### Religion

All the study participants were Christians but from different denominations. Some of those who were Roman Catholics had challenges related to cryopreservation or discard of unused embryo. Respondent No. 3 said:

As a Roman Catholic, I told them to transfer all my embryos irrespective of the status because I did not want any to be frozen or destroyed. [forty-eight years old laboratory scientist, Igbo]

Similarly, respondent No. 6 said:

Although the doctor explained the benefit of freezing the excess embryos, my spirit did not accept it because I believe it is a sin. [forty-four years old Pharmacy Rep, Igbo] However, some of the study participants were happy with the availability of embryo cryopreservation. Respondent No. 7 said:

I was told to pay one hundred thousand naira extra if I need my embryos preserved. Thank God I did because it failed the first time. I was glad there was no need to go through the whole process again. I had four babies at my second attempt (She smiles happily). [forty-three years old businesswoman, Hausa]

### Cost

All the study participants agreed that ART is expensive and not within the reach of the average Nigerian woman. According to respondent No. 6,

I paid an initial deposit of seven hundred and fifty thousand naira by bank-draft, but also paid for my investigations and drugs. By the time of the embryo transfer, it was one and a half million naira. I still had to cover the drugs given after embryo transfer, including investigations. [forty-four years old Pharmacy Rep, Igbo]

Some resorted to loans or seeking help from relatives or community members. The high cost of the facilities is one of the reasons female clients present at advanced age thereby reducing the success rate of the procedure. Respondent No. 6 said:

My husband and I were able to access ART after eleven years of infertility with the support of church members. Even though we heard of ART from relatives who were successful, we kept hoping that conventional treatment and prayers will help us. We could not afford the cost of treatment as we were told it runs into hundreds of thousands of naira.” [forty-four years old Pharmacy Rep, Igbo]

### Challenges with Roads

Some of the participants had challenges with getting to and from the ART clinics due to bad roads. Some felt that miscarriages following embryo transfer occur due to the bad roads and distance challenges to the centre they accessed. According to respondent No. 4,

The road to the facility where I had my treatment was bad, and I know many women who had miscarriages after their embryo-transfer. I believe the bad road may have been a factor. [thirty-seven years old businesswoman, Igala]

This was corroborated by respondent No. 3:

I had my procedure in another state and had to travel back home on bad roads. I was successful at my third attempt and that was because we stayed in a nearby hotel for three months after the transfer. [forty-eight years old laboratory scientist, Igbo]

### Information disclosure

A major challenge identified by many of the clients was lack of adequate information on what the procedure entailed and its adverse effects. Respondent No. 1 said:

It was when I went to the UK that the risks were explained to me. I had never heard of hyperstimulation of the ovaries until it happened to me. [fifty-two years old laboratory scientist, Igbo]

She also added:

I had severe abdominal pains during the drug injection phase and was rushed back to the ART clinic. That was when I was told that it is hyperstimulation of my ovaries. I was referred to the intensive care unit of a teaching hospital and I paid for my treatment. [fifty-two years old laboratory scientist, Igbo]

Similarly, respondent No. 5 said:

Though the procedures were explained, and we were told that there is fifty-percent chance of success, the risks were not mentioned. [thirty-eight years old businesswoman, Igbo]

### Shared decision-making

The respondents also noted that during the general counselling sessions, they were told that a maximum of five embryos were to be transferred but their opinions were not sought as to the number to be transferred during the transfer stage, nor information on number of eggs retrieved or embryos made. According to respondent No. 4,

During counselling, we were told that they put a maximum of five embryos, but I was not told how many embryos were transferred during the embryo-transfer stage. [thirty-seven years old businesswoman, Igala]

Similarly, respondent No. 7 said:

Five embryos were transferred during my successful cycle, but the total number of eggs retrieved, or embryos made was never discussed with my husband and I. [forty-three years old businesswoman, Hausa]

This was further corroborated by respondent No. 2 who said:

It was while I was on the couch during the embryo transfer stage that a transcript was handed over to me indicating the number of eggs retrieved, embryos made and number being transferred, which I was only able to look at after the procedure. [thirty years old immigration officer, Igbo]

### Gendered Suffering

Most of the respondents had difficulties accessing ART due to lack of support from their spouses and the unequal reproductive burden imposed by society on the woman. According to respondent No. 4,

My husband refused to give consent for us to access ART because he thought they are abnormal children. I had to tell him about his niece, who he had no knowledge was conceived by the same technology. He agreed the child is normal, and that was when he then agreed we see the ART specialist. [thirty-seven years old businesswoman, Igala]

Similarly, respondent No. 2 said:

You don't tell anyone you had your baby through ART or else they say ‘agbanyelu gi nwa n'afo’ (they injected baby into your uterus) [thirty years old immigration officer, Igbo]

The infertility burden on the woman was further corroborated by respondent No. 7, who said:

For me I don't really care about the cost. I was prepared to pay above what was requested because it is better than people insulting you that you cannot bear children. [forty-three years old businesswoman, Hausa]

### Psychological challenges

Many of the respondents complained that they suffered a high level of anxiety and fear during the assisted conception process. According to respondent No. 8,

The emotional burden on the woman is tremendous. I had seven attempts before success (shakes her head). [forty-three years old medical practitioner]

This was corroborated by respondent No. 6:

I was depressed all through the process. Luckily, the transfer was successful the first time. Only those who have gone through it can imagine what women go through when they have a failed cycle. [forty-four years old Pharmacy Rep, Igbo]

## Discussion

Assisted reproductive technology has been hailed as an answer to the problem of infertility worldwide and millions of infertile individuals and couples have benefited from this technology. However, it is known to be associated with numerous challenges, more so in developing countries where the prevalence of poverty and ignorance remain high.

### ART access issues

Reasons for lack of early access for assisted reproductive technology among our study clients varied from ignorance of the availability of the technology, not being referred on time by the family physician or conventional gynaecologist, religious/cultural bias, and lack of funds.

Results from this study indicate that information on which centre to access for treatment is mostly based on recommendation from friends or relatives. This was the experience of our participants. This agrees with findings in other Nigerian studies 15, 18, 20. While this has its advantages, it could lead to clients being misled by unscrupulous individuals. This therefore emphasizes the importance of accreditation of ART centres by Government. Though some big centres have websites in which the range of services they offer are advertised, it is against medical ethics to advertise about the skill of personnel and success rate 21. Prior to the AFRH-ART guideline, many centres put up promotional adverts and fliers in which they offer discounted rates for the ART procedures to increase their clientele. However, this has been banned under the guideline as it could lead to loss of respect and trust for the medical profession by the public. Nigeria is also yet to have an organized national data that is available to the populace on success rates of different centres. This contrasts with the developed nations where information on centres to access is available on the internet, in addition to referral from general practitioners and gynaecologists.

Some of the study clients were discouraged by their spouses and relatives because they believed they should try the conventional methods for a longer time since they were still young. More than half of our study clients accessed ART above the age of forty years. It is known that the younger a woman is, the higher the likelihood of success of ART, both in terms of using her own eggs, increased rate of embryo implantation and reduction in complications. As women age, their ability to produce healthy eggs for healthy embryo formation decreases. Another client reported that her husband initially did not support their use of ART because he thought offspring from the technology were abnormal. This is probably from fear of stigmatization as noted by Jegede & Fayemiwo (2010) 18, which explains why those who are successful with ART keep it secret 22 from others especially family members. Increased awareness and educational programs are necessary for the benefit of the society to prevent these sociocultural limitations to access.

Some of the clients also reported being discouraged by their gynaecologists whose responsibility it is to get them the needed help for their condition. This stems from physician conflict, and calls for disclosure of conflict of interest to the clients. Conscientious objectors who have sound personal or religious convictions should be allowed to refrain from the provision of ethically sensitive services such as abortion and some ART practices 23. This needs to be balanced, however, by restraint from judgmental or negative behaviour toward the client seeking these services. The clients should be referred to other physicians where they can access the care they need. This further emphasizes the importance of a guideline mandating physician-disclosure of any conflict to patients, as well as state provision of means of accessing the information on ART service providers without having to go through general physicians or gynaecologists if the clients so choose.

All our clients were Christians, though from different denominations. Half of our participants were Roman Catholics, despite the Church's stand forbidding all forms of ART. This shows that people do not always follow the tenets of the religion they profess. The great majority of the population coming from a Catholic upbringing (84.4% claim to be Catholic) do not follow Catholic teachings as noted in an Argentinian study 24. This phenomenon is not unique to ART. In contraception, the attitudes are identical. For example, though abortion laws are highly restrictive, illegal abortions are carried out continuously. These facts speak of hypocrisy and a double moral standard 25.

Notwithstanding the above findings, religion remains a major limitation to the choice of ART. Some of the Roman Catholics insisted that all their embryos should be transferred irrespective of number and status. This is to prevent their (embryos) anticipated loss of dignity following cryopreservation and the thawing process, or destruction which is seen as an abortion and thus a sin. These women are conscientiously forced to request for multiple embryo transfer despite the increased maternal and fetal morbidity and mortality that may result 26. In addition, these women may have to submit to more frequent hormones and medication intake with the inconvenience and harm this may entail should there be cycle failure since there will be no cryopreserved embryo to fall back on. The service providers are caught in the dilemma of choosing between respect for client's true informed consent, and disrespect for client's autonomy, to avoid harm to the client. The ART practitioners can do well to use low dose stimulation protocols 27 and retrieval of just the allowed number of oocytes which will be in line with good ethical clinical practice 28, despite the informed consent of the clients.

The average cost of ART ranged from one and a half to three million naira depending on whether the infertile couple's gametes or donor's gametes are used as reported by the clients. Though the cost is high worldwide, it is more so in Sub-Saharan Africa including Nigeria where poverty is endemic 29. In the absence of health insurance, people pay out of pocket and very few can afford it. Many of the clients saved money over prolonged periods (which is one of the reasons they presented at advanced age of female partners), took loans, sold properties, or accepted support from relatives and churches. ART provides an excellent example of stratified reproduction where assisted reproduction is restricted to the global elites, leaving the infertile poor with limited access due to the cost. Looking at ART through the prism of reproductive rights, in addition to a person's right to control his/her fertility, one should also exercise his/her right to facilitate fertility when fertility is threatened. For millions of couples facing tubal or male infertility, facilitation of fertility may require resort to ARTs. Achieving full reproductive rights around the globe means achieving access to IVF and related technologies. In the words of Mahmoud Fathalla (2002) 29, family planning must also mean planning for families. The National Health Insurance Scheme (NHIS) does not cover routine infertility treatment, not to mention assisted conception services.

However, considering that procreation and parenting are of such central importance to an individual's identity and life goals, medical insurance should pay for infertility treatment 30. Regulation of ART will in-cooperate this requirement, thereby protecting those who need this technology irrespective of their financial status.

### Number of transferred embryos

The participating clients in the study confirmed that the centres they accessed transfer five embryos as a matter of protocol though the AFRH-ART guideline recommends a maximum of three 14. A study in Port-Harcourt, Nigeria found that as many as 94.4% of clients also explicitly requested transfer of two (66.6%) or more than two (27.8%) embryos 31. In addition, Africans including Nigerians see multiple pregnancy as a blessing rather than as a risk factor 32. This is confirmed by the client in this study who was so happy with delivery of quadruplets. This has ethical implications as there is a high risk of higher order multiple pregnancies with its attendant complications of maternal and fetal morbidity and mortality. The ART client who had quadruplets following transfer of five embryos was faced with maternal complications of hypertension in pregnancy, pre-eclampsia, gestational diabetes, iron-deficiency anaemia, antepartumnd postpartum haemorrhage, prolonged admission in hospital, and increased operative delivery, as well fetal complications of intrauterine demise of one or more of the fetuses, preterm birth, and prematurity 33.

The Hippocratic Oath, usually sworn by doctors on graduation cover several important ethical issues between them and their patients. The oath serves as a contract for doctors to work for the benefit of the health of the public, as well as preventing exploitation of the patient. For clinics to increase their success rates, they transfer multiple embryos despite knowing the risks implied, while exploiting the desperation of clients for successful pregnancies. The practitioners are supposed to work within the safest boundary since they are better informed of the consequences of higher order multiple pregnancies irrespective of cost of the procedure or the need of clients for multiple gestations. Facilities for fetal reduction in Nigeria is lacking and even in centres where it may be present, any attempt at this is seen as abortion and greatly frowned at.

The ethical principles of beneficence (moral obligation to act for the benefit of others) and non-maleficence (‘Primum Non Nocere’-- obligating one to abstain from causing harm to others) should form the basis of the response of the practitioners to the clients despite their explicit or perceived need for multiple pregnancies 28. An unbiased proper information on the risks involved is likely to help them make an informed choice. While respect for patient autonomy is a central constituent of clinical ethics, it has only prima facie standing and competing moral considerations could sometimes override this principle. When patients request for medically inappropriate treatment, beneficence-based considerations and the virtue of professional integrity should draw a limit to patient's autonomy.

### Information disclosure

Internationally, informed consent process for ART procedures consists of a discussion with the clients about the procedures, outlining the risks and effects, supplemented by written materials to help them make up their minds on whether to proceed with the procedure or not. Our clients claimed that they were given minimal information on the procedure. They all agreed that they were counselled to expect fifty percent chance of success. They noted that they were told that it is God that will give them a child during the counselling session, and the doctors told them immediately after embryo transfer that the rest is up to God.

With regards to the risks involved, one client claimed she developed ovarian hyperstimulation syndrome (OHSS) and was only told about the complication when she presented with the emergency. Despite signing written consent for the procedures, most of the ART clients did not fully understand the effect of the drugs or procedures on their health. In the absence of adequate comprehension, which negates voluntariness, there is no legal authorization for the procedures. The clients were only interested in the pregnancy, mostly due to family and societal pressure. The clients could not have consented to the procedures if they did not understand the risks of the procedure.

The heart of the informed consent process is the explanation of the procedure and the benefits, risks, and alternatives to the proposed treatment. The forms likely will explain in considerable detail the myriad risks of the treatment, including any medications prescribed, to the patient (or donor or surrogate), as well as to any children resulting from the treatment. Informed consent does not require the physician to disclose every conceivable risk, no matter how remote or unlikely. But risks of serious adverse outcomes should generally be disclosed.

Most of the clients complained that they had no idea as to the number of eggs retrieved from their ovaries, nor the number of embryos transferred. Only one of the clients was given a transcript of the eggs retrieved and embryos transferred after the embryos had been transferred. This is against the principle of shared decision-making which is the current international standard of care. Though the ART specialists have a better knowledge of what is best for the clients, paternalism is no longer acceptable in current medical practice.

### Gendered Suffering

According to one of the ART clients, “women are ready to sell their souls to the devil and even sleep with other men to have a child regardless of risks involved”. This explains why many of the study clients went through multiple cycles of ART until they were successful. In Nigeria, as happens in other Sub-Saharan African countries, a child is the ultimate blessing that binds and confirms a marital union. In addition, a woman is only deserving of the name “woman” and most importantly “wife” when she has been seen to have delivered a child. Within childless marriages, women are likely to be victims of domestic violence 34 and may also endure various forms of emotional and verbal abuse from husband and husband's family members. It is not therefore surprising that women are ready to try anything to have a child no matter how detrimental it is to their health. They are more likely than fertile women to be exposed to HIV from extramarital attempts to conceive 35.

It is not unusual for a husband to abandon his wife once no child is forthcoming from their union. In societies where polygyny is allowed, men may take second wives rather than separating or divorcing their wives. According to the forty-seven country DHS survey, in Kenya and Nepal, men whose first wives are childless, are twenty percent and nineteen percent more likely to take second wives respectively 35. These women are taunted about their barrenness and lack of femininity 36. They may be turned away from life-cycle rituals involving other women and their children, accusing them of casting an evil eye on other women's children from uncontrollable envy 37. Without children in the household, severe difficulties in achieving security at old age results, especially for elderly women 38.

Adoption, while an option in infertility is not yet fully acceptable in Africa and remain chaotic in Nigeria. The adopted child is usually discriminated against 39 by the extended family of the man and may not be given any inheritance unless the adoptive father has a Will or buys property specifically in the name of the child. Women go as far as pretending to be pregnant, involving intake of various injections with the intent to deceive neighbours and family while waiting for their adoption process to mature. The husband of one of the study clients had no knowledge that his favourite niece was born with the help of ART and was only intimated by the study client as a leverage to get him to consent to ART for their own infertility problem. According to many of the study clients, women with IVF pregnancies are taunted that their babies were artificially injected into their bodies. Hence, despite the widespread use of ART, it is often associated with feelings of shame. Such feelings often lead the ART parents to avoid open discussion about their infertility treatments. This was reiterated by one of the study participants that when the general society gets to know about the inability of a couple to have children and the resort to assisted methods (IVF), they may be stigmatized, call their children abnormal or unnatural, and therefore may not be accepted by members of the kinsmen as part of their family members. Therefore, IVF technology is still masked in secrecy, stigma, misconceptions, and religious sentiments in Nigeria 15.

This has fuelled the stories of cryptic pregnancy, prevalent in the South-eastern States of Nigeria. This situation usually involves a woman looking for the fruit of the womb who consults a facility where she is told that she is already pregnant, and that the pregnancy cannot be detected by doctors and ultrasound scans because the pregnancy is hiding at the back of the abdomen or the spines. Hormonal drugs are sometimes given to stop menstruation and make the client bloated up. Apparently anytime a baby is available, the client is called up for a phantom labour and delivery. A higher fee is charged for male babies 40. It is a mafia of illegal buying and selling of children 41.

The tremendous anxiety the infertile couples go through, in their effort to fulfil their societal-given roles cannot be quantified. Hence, it is only a matter of justice that every conceivable effort is made by Government to make ART accessible while clamping down on illegal practitioners who exploit the vulnerability of these members of society.

## Conclusion

Our study findings show that the cost of ART remain prohibitive and unaffordable by most infertile couples. This is unacceptable especially from the prism of reproductive rights, which must include the right to assist fertility when fertility is threatened, in addition to the right to control high fertility 3. There is therefore an urgent need for ART to be offered by more government owned establishments with subsidization of services, as well as its inclusion in the National Health Insurance Scheme 42.

The five embryos routinely transferred into the uterus of the study clients is greater than the maximum of three, recommended by the AFRH-ART professional guideline. Due to the lack of legal enforceability of the guideline across the country, with the exception of Lagos State, the practitioners are left to work according to their personal moral codes and consciences, and not everyone has a well-formed conscience 43. This has led to an increase in the risk of inappropriate practice. Therefore, the calls for all stakeholders to ensure the passage of the Nigeria Assisted Reproductive Technology Bill, which has been in the House of Assembly since 2012 44 cannot be overemphasized. The passage of this Bill will ensure a universal standard of ethical practice, with penalties for violation.

In addition, a national database of accredited ART clinics made publicly available and accessible to clients will provide the needed information on how to access professionally qualified practitioners.

The effect of the enormous psychological trauma and social stigma resulting from involuntary childlessness as confirmed by our findings can be ameliorated by institution of integrated reproductive health programs, educating both males and females at the community level, on the causes of infertility and the negative impact of various myths and sociocultural beliefs on the woman and society-at-large 43.

## Study Limitation

Though the number of female ART clients studied were few, the exploratory research design ensured that their experiences were thoroughly assessed. The clients accessed care from four of six geopolitical zones of the country hence the results may not be totally generalizable to the whole country. This study also explored only the female clients and a study on the challenges experienced by male ART clients is worth considering.
